# Supplementary material for: Implementing quantum dimensionality reduction for non-Markovian stochastic simulation
Source: Nat Commun. 2023 May 6;14:2624. doi: 10.1038/s41467-023-37555-0 (PMC10164178; doi:10.1038/s41467-023-37555-0)
Supplement: Supplementary file 1 — Supplementary Information [file 41467_2023_37555_MOESM1_ESM.pdf]

Supplementary Material – Implementing quantum dimensionality reduction for non-Markovian stochastic simulation

Kang-Da Wu,[1, 2, 3] Chengran Yang,[4] Ren-Dong He,[1, 2, 3] Mile Gu,[5, 4, 6]
Guo-Yong Xiang,[1, 2, 3] Chuan-Feng Li,[1, 2, 3] Guang-Can Guo,[1, 2, 3] Thomas J. Elliott,[7, 8, 9]
[1]*CAS Key Laboratory of Quantum Information, University of Science and Technology of China, Hefei 230026, People's Republic of China*
[2]*CAS Center For Excellence in Quantum Information and Quantum Physics, University of Science and Technology of China, Hefei, 230026, People's Republic of China*
[3]*Hefei National Laboratory, University of Science and Technology of China, Hefei 230088, People's Republic of China*
[4]*Centre for Quantum Technologies, National University of Singapore, 3 Science Drive 2, Singapore 117543, Singapore*
[5]*Nanyang Quantum Hub, School of Physical and Mathematical Sciences, Nanyang Technological University, Singapore 637371, Singapore*
[6]*MajuLab, CNRS-UNS-NUS-NTU International Joint Research Unit, UMI 3654, Singapore 117543, Singapore*
[7]*Department of Physics & Astronomy, University of Manchester, Manchester M13 9PL, United Kingdom*
[8]*Department of Mathematics, University of Manchester, Manchester M13 9PL, United Kingdom*
[9]*Department of Mathematics, Imperial College London, London SW7 2AZ, United Kingdom*

S1. QUANTUM MODELS OF PMD PROCESSES

Recall from the main manuscript that quantum models of PMD processes with period N consist of a set of N memory states $\{|\sigma_n\rangle\}, n \in [0..N-1]$, and a pair of Kraus operators $\{A_0, A_1\}$ satisfying

$$A_0|\sigma_n\rangle \propto |\sigma_{n+1 \mod N}\rangle$$
$$A_1|\sigma_n\rangle \propto |\sigma_0\rangle. \quad (1)$$

These Kraus operators must further satisfy the completeness relation $A_0^\dagger A_0 + A_1^\dagger A_1 = \mathbb{1}$. We now show how these can be constructed for any PMD process with all memory states encoded within a 2-dimensional Hilbert space. We remark that the set of Kraus operators that manifest the requisite statistics may not be (and in general, will not be) unique; nevertheless, we need only determine one such set that does indeed yield valid statistics.

The periodicity of the model mandates that N applications of A_0 must return the initial state, such that

$$A_0{}^N = \frac{1}{\eta^N}\mathbb{1} \quad (2)$$

for some real, positive number η. We remark that when the environment manifesting the Kraus operators is assumed to only be measured/decohere in the basis associated with their labels, the Kraus operators can freely be multiplied by a complex phase factor without observable physical effect; in the context of our work, this corresponds to the output qubits only being measured in the computational basis. We can use this symmetry to limit our attention without loss of generality to ηA_0 with eigenvalues 1 and $\exp(i\phi)$ with associated eigenvectors

$$|Z_0\rangle = \begin{bmatrix}1\\0\end{bmatrix} \quad \text{and} \quad |Z_1\rangle = \begin{bmatrix}\alpha\\1\end{bmatrix} \quad (3)$$

where $\phi = 2m\pi/N$, $m \in \mathbb{Z}$, and α is a number, assumed real. Thus, A_0 has the form,

$$A_0 = \frac{1}{\eta}\begin{bmatrix}1 & \alpha(e^{i\phi}-1)\\0 & e^{i\phi}\end{bmatrix}. \quad (4)$$

We shall proceed to consider explicitly only the case where $m = 1$.

Consider the singular value decomposition of A_0:

$$A_0 = \xi_0|u_0\rangle\langle v_0| + \xi_1|u_1\rangle\langle v_1|, \quad (5)$$

where $\{|u_j\rangle\}$ and $\{|v_j\rangle\}$ each form orthonormal bases, and ξ_j are the singular values. These singular values can be deduced to be

$$\xi_{0,1}^2 = \frac{1}{\eta^2}\left(1 + \frac{\gamma}{2} \pm \frac{1}{2}\sqrt{\gamma^2 + 4\gamma}\right), \quad (6)$$

where $\gamma = 4\alpha^2 \sin^2(\phi/2)$.

Meanwhile, we can always cast A_1 in the form

$$A_1 = \zeta|\sigma_0\rangle\langle w|, \quad (7)$$

for some vector $|w\rangle$ and number ζ of at most unit magnitude. Since the Kraus operators $\{A_0, A_1\}$ must satisfy the completeness relation, together this requires

$$\zeta^2|w\rangle\langle w| + \xi_0^2|v_0\rangle\langle v_0| + \xi_1^2|v_1\rangle\langle v_1| = \mathbb{1}. \quad (8)$$

A (not necessarily unique) valid solution to this is given by

$$|w\rangle = |v_1\rangle$$
$$\xi_0 = 1$$
$$\zeta^2 + \xi_1^2 = 1. \quad (9)$$

This implies

$$\eta^2 = 1 + \frac{\gamma}{2} + \frac{1}{2}\sqrt{\gamma^2 + 4\gamma}. \quad (10)$$

Let us now express the first memory state $|\sigma_0\rangle$ is in terms of the eigenvectors of A_0:

$$|\sigma_0\rangle = \beta_0|Z_0\rangle + \beta_1|Z_1\rangle \quad (11)$$

for some coefficients β_0 and β_1, assumed real. Normalisation of the state constrains the coefficients, such that

$$(\beta_0 + \beta_1\alpha)^2 + \beta_1^2 = 1. \quad (12)$$

By applying A_0 to $|\sigma_0\rangle$ n times, it can then be deduced that (neglecting normalisation)

$$|\sigma_n\rangle \propto A_0^n|\sigma_0\rangle \propto \beta_0|Z_0\rangle + \beta_1 e^{in\phi}|Z_1\rangle. \tag{13}$$

This evolution will output the statistics of a renewal process with survival probability $\Phi(n)$ given by

$$\begin{aligned}\Phi(n) &= |\langle\sigma_0|A_0^{\dagger n} A_0^n|\sigma_0\rangle| \\ &= \frac{1}{\eta^{2n}}(|\beta_0 + \alpha\beta_1 e^{in\phi}|^2 + \beta_1^2) \\ &= \frac{1}{\eta^{2n}}(1 - 2\alpha\beta_0\beta_1(1-\cos(n\phi))), \\ &= \frac{1}{\eta^{2n}}\left(1 - 4\alpha\beta_0\beta_1\left(1 - \sin^2\left(\frac{n\phi}{2}\right)\right)\right),\end{aligned} \tag{14}$$

where we have used Eq. (12) to reach the penultimate line.

This can be seen to be of the form of a PMD process, with $V = 4\alpha\beta_0\beta_1$, $\Gamma = 1/\eta^2$, and $\theta = \phi/2$. Together with Eqs. (10) and (12), these equations can be solved to deduce the appropriate values of (α,β_0,β_1) to construct a quantum model of any given PMD process.

Returning to our exposition on quantum models in the main text, we remark that any pair of Kraus operators $\{A_0, A_1\}$ satisfying the completeness relation (as the above do by construction) can be implemented through the use of a joint unitary interaction between the system on which they act and a qubit ancilla. This ancilla then corresponds to the output qubit of the model. Note also that we have ensured that all N quantum memory states inhabit the Hilbert space spanned by a qubit by construction, as the (complete) Kraus operators act only on the space of a single qubit.

S2. EMBEDDING QUANTUM EVOLUTION AS A QUANTUM WALK

The simulation module of our implementation evolves the initial memory state to perform a simulation of two timesteps of the given PMD process. As noted in the main manuscript, this is achieved by embedding the desired evolution within a photonic quantum walk. Such a walk consists of two degrees of freedom: the position of a 'walker', and a 'coin'. The position takes values $p \in \mathbb{Z}$, and is represented by the spatial path of the photon. The coin takes on two discrete values $\{0, 1\}$ and is encoded in the polarisation of the photon. Each step of the walk comprises of two unitary evolutions acting on the joint coin-position system $\mathcal{H}_c \otimes \mathcal{H}_p$. The first is a position- and step-dependent conditional evolution of the coin state:

$$C(k) = \sum_p C(p,k) \otimes |p\rangle\langle p|, \tag{15}$$

where $C(p,k)$ is a unitary evolution acting on the coin, and k indexes the walk step. The second is a coin-conditional translation operation that shifts the position of the walker:

$$\mathcal{T} = \sum_p |0\rangle\langle 0| \otimes |p+1\rangle\langle p| + |1\rangle\langle 1| \otimes |p-1\rangle\langle p|. \tag{16}$$

The total walk consists of K such steps, given by a full evolution operator $\mathcal{U}(K-1)\ldots\mathcal{U}(1)\mathcal{U}(0)$, where $\mathcal{U}(k) := \mathcal{T}C(k)$. By appropriate engineering of the conditional coin evolutions $C(p,k)$, we are able to realise the desired evolution of our model's simulation stage.

Our simulation requires $K = 3$ steps for each walk, to implement one timestep of the model simulation. First consider a singular value decomposition of the two Kraus operators $\{A_j\}$:

$$\begin{aligned}A_0 &= U_0 D_0 V_0, \\ A_1 &= U_1 D_1 V_1,\end{aligned} \tag{17}$$

where the U_j and V_j are unitary operators, and the D_j are diagonal matrices. From the completeness relation of Kraus operators it follows that

$$V_1^\dagger D_1^2 V_1 = \mathbb{1} - V_0^\dagger D_0^2 V_0 = V_0^\dagger(\mathbb{1} - D_0^2)V_0, \tag{18}$$

where we have used the unitarity of V_0. A solution to the above is given by (in close analogy to Eq. (9))

$$\begin{aligned}V_0 &= V_1 \\ D_0^2 &= \mathbb{1} - D_1^2.\end{aligned} \tag{19}$$

Putting this together with the details of the theoretical model construction as described in the previous section, we obtain

$$\begin{aligned}V_0 = V_1 &= |0\rangle\langle v_0| + |1\rangle\langle v_1| \\ D_0 &= \begin{pmatrix} 1 & 0 \\ 0 & \xi_1 \end{pmatrix} \\ D_1 &= \begin{pmatrix} 0 & 0 \\ 0 & \zeta \end{pmatrix} \\ U_0 &= |u_0\rangle\langle 0| + |u_1\rangle\langle 1| \\ U_1 &= |\sigma_0\rangle\langle 1|.\end{aligned} \tag{20}$$

These can then be implemented using the following conditional coin evolutions:

$$\begin{aligned}&C(0,1) = V_0 \\ &C(1,2) = \begin{pmatrix} 0 & 1 \\ 1 & 0 \end{pmatrix} \qquad C(-1,2) = \begin{pmatrix} \xi_1 & \zeta \\ \zeta & -\xi_1 \end{pmatrix} \\ &C(0,3) = U_1 \qquad C(-2,3) = U_0.\end{aligned} \tag{21}$$

The topology of this is shown in Fig. 3. Note that in quantum optics any unitary can be implemented on a polarisation-encoded qubit using a Q-H-Q (quarter-half-quarter wave plate) configuration; thus, in our implementation V_0 is realised via three wave plates. The combination of two BDs (effecting the translation operation) and two HWPs realise D_0 and D_1; the upper path evolves under D_0 and the lower D_1. Outcome 0 is followed by a HWP and a QWP, realising U_0, while a Q-H-Q acts as U_1. The total walk thence implements the desired unitary for one timestep of our simulation.
